# Supplementary material for: Inkjet-Printed Phospholipid Bilayers on Titanium Oxide Surfaces: Towards Functional Membrane Biointerfaces
Source: Membranes (Basel). 2022 Mar 25;12(4):361. doi: 10.3390/membranes12040361 (PMC9030265; doi:10.3390/membranes12040361)
Supplement: Supplementary file 1 [file membranes-12-00361-s001.zip › membranes-1649887-supplementary.pdf]

Supplementary Materials

Inkjet-Printed Phospholipid Bilayers on Titanium Oxide Surfaces: Towards Functional Membrane Biointerfaces

Sigalit Meker [1], Oded Halevi [2], Hokyun Chin [1], Tun Naw Sut [3], Joshua A. Jackman [3], Ee-Lin Tan [1], Michael G. Potroz [1] and Nam-Joon Cho [1,*]

[1] School of Materials Science and Engineering, Nanyang Technological University, 50 Nanyang Drive,
Singapore 637553, Singapore; sigi.meker@gmail.com (S.M.); full.ahead.hogyun@gmail.com (H.C.); eelintan05@gmail.com (E.-L.T.); mikepotroz@gmail.com (M.G.P.)

[2] The Institute of Chemistry, The Hebrew University of Jerusalem, Jerusalem 91904, Israel; odedqs@gmail.com (O.H.)

[3] School of Chemical Engineering and Translational Nanobioscience Research Center, Sungkyunkwan
University, Suwon 16419, Korea; suttunnaw@skku.edu (T.N.S.); jjackman@skku.edu (J.A.J.)

* Correspondence: njcho@ntu.edu.sg

DOCP lipid bilayers are tethered to TiO_2 surface

To confirm that DOCP bilayers are tethered to the TiO_2 surface via covalent bonds, the bilayers were washed with absolute ethanol while monitored with the QCM-D. Upon ethanol wash, only the upper leaflet of the lipid bilayer was removed, thus supporting that the lower leaflet was tethered to the surface.

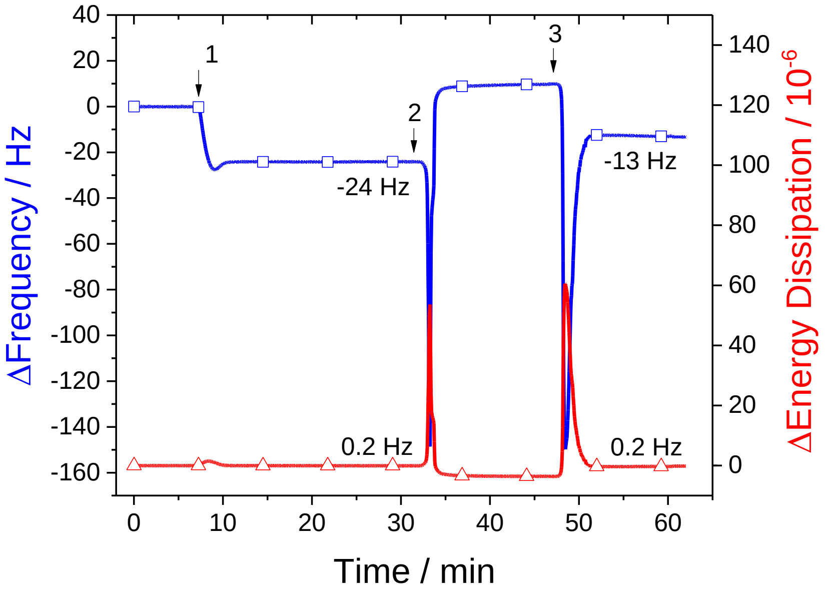

Figure S1. Characterization of DOCP lipid upper leaflet removal by ethanol treatment. DOCP lipid vesicles were adsorbed and ruptured on the surface, to form bilayers (Δf = -24 Hz; ΔD = 0.2 x 10^{-6}) (1). Then absolute ethanol was added for 15 min. (2), followed by buffer wash for 10 min. (3). Upon ethanol and buffer rinse, frequency and dissipation energy shifted to values that fit monolayer formation, Δf = -13 Hz; ΔD = 0.2 × 10^{-6}. This implied that the lower leaflet of the DOCP bilayer was chemisorbed and therefore was not affected by the ethanol rinse [1].

References

1. Wang, F.; Liu, J. A stable lipid/TiO_2 interface with headgroup-inversed phosphocholine and a comparison with SiO_2. *J. Am. Chem. Soc.* **2015**, *137*, 11736-11742.
